# Reliability Analysis for Unrepairable Automotive Components

**DOI:** 10.3390/ma14227014

**Published:** 2021-11-19

**Authors:** Dariusz Ulbrich, Jaroslaw Selech, Jakub Kowalczyk, Jakub Jóźwiak, Karol Durczak, Leszk Gil, Daniel Pieniak, Marta Paczkowska, Krzysztof Przystupa

**Affiliations:** 1 Faculty of Transport and Civil Engineering, Institute of Machines and Motor Vehicles, Poznan University of Technology, Piotrowo 3, 60-965 Poznan, Poland; dariusz.ulbrich@put.poznan.pl (D.U.); jakub.kowalczyk@put.poznan.pl (J.K.); marta.paczkowska@put.poznan.pl (M.P.); 2 Solaris Bus & Coach S.A., Obornicka 46, 62-005 Owinska, Poland; jakub.jozwiak@solarisbus.com; 3 Department of Biosystems Engineering, Faculty of Environmental and Mechanical Engineering, Poznan University of Life Sciences, Wojska Polskiego 50, 60-627 Poznan, Poland; karol.durczak@up.poznan.pl; 4 Department of Mechanics and Machine Building, University of Economics and Innovations in Lublin, Projektowa 4, 20-209 Lublin, Poland; leszek.gil@wsei.lublin.pl; 5 The Main School of Fire Service, Faculty of Safety Engineering and Civil Protection, Slowackiego 52/54, 01-629 Warsaw, Poland; dpieniak@sgsp.edu.pl; 6 Department of Automation, Lublin University of Technology, Nadbystrzycka 6, 20-618 Lublin, Poland

**Keywords:** reliability, failure analysis, automotive

## Abstract

The analysis of the reliability parameters of a technical object and the determination of the change in the reliability of the object over time, requires the knowledge of the functional characteristics and reliability parameters of the elements included in a system. On the basis of the failure data of the selected element of the object, in this case the vehicle, it is possible to determine the average working time to failure of the element and the appropriate form of distribution that characterizes the reliability and durability parameters of the tested element. The main purpose of the research presented in the article was to develop a method of assessing the reliability of an electronic component of a vehicle-a boot lid contactor. This paper also presents three possible methods of repairing the boot lid contactor (sealing the housing with adhesive with better way, replacing the element with a new one or the most time-consuming solution, changing the shape of the boot lid). The authors also decided to determine the reliability and cost parameters that will allow preventive replacement of this element. The tests were carried out on a fleet of 61 vehicles of the same model, but with different body structures. Contactor failures were reported in 41 cases, of which 29 were in the hatchback construction and 12 in the estate type. The analysis of the distribution selection for the tested part of the passenger car-the boot lid contactor-was performed using the Likelihood Value (LKV) test to determine the rank of distributions. Also the maximum likelihood (MLE) method was used to estimate the distribution parameters. The three-parameter Weibull distribution was the best-fitted distribution in both cases. It was clearly defined that one model of car with two different types of body have vastly different reliability characteristic. Based on the reliability characteristic and parameters, the appropriate preventive actions can be taken, minimizing the risk of damage, thus avoiding financial losses and guaranteeing an appropriate level of vehicle safety.

## 1. Introduction

In the reliability analysis of technical objects, various probability distributions can be used for modeling damage data [1,2,3]. Nevertheless, the most commonly used probability distributions are the normal, exponential, Weibull or gamma ones [4,5,6]. In the research results presented in this paper, for the reliability analysis of the boot lid contactor, apart from the above distributions, other, less frequently used distributions were used, the quality of which, for the analyzed case, is better than the usually used distributions. Less frequently used distributions include the log-normal distribution, the generalized gamma distribution, the logistic distribution, the log-logistic distribution, and the Gumbel distribution. The parameters of these distributions can be estimated using analytical methods as well as numerically using specialized IT tools [7,8].

Evaluation of the parameters of a distribution includes data modeling that requires the determination of the best-fit distribution and the estimation of the parameters of this distribution (shape, scale, position). Various methods of parameter estimation are used, including both numerical and graphical methods [9,10,11]. The most frequently used methods include the method of moments, the method of maximum likelihood, the method of least squares, fitting in distribution grids, the method of correlation coefficient of the probability plot (PPCC) and others [12,13,14].

Based on the damage data and the assumed estimation method, the parameters are estimated (shape, scale, location) for selected families of probability distributions [15,16,17]. Based on estimated different distributions, it is possible to indicate among them the best suited to empirical data in terms of the least squared sum of deviations [18,19].

The main purpose of the research presented in the article was to develop a method for assessing the reliability and costs related to corrective maintenance of the boot lid contactor of a motor vehicle. The tests were carried out on a fleet of 61 vehicles of the same model but with a different body structure. The analysis used three factors the modified Kolmogorov-Smirnov (K-S) statistics, the statistics of the mean absolute deviation of the hypothetical from the empirical distribution (rho) and the statistics calculated on the basis of the logarithmic likelihood function (LKV) to determine the best fitted distribution.

The proposed methodology for identifying the contactor’s lifetime takes into account all available data on vehicle operating times, expressed in kilometers (km) according EN50126-1 Standard. This includes the case where a given item is fit at the time the research is discontinued, and the lifetime of such an item is called right-censored. Right-censored data was used in the research. Right-censored data also called suspended data, is composed of units that did not fail during the test. They are considered “still alive” as their failure time has not yet occurred, though it is expected to occur at some point in the future. The method of preparing statistical data on the basis of the operational data was developed in the works [20,21,22]. All vehicles were operated by drivers, representatives of one company. Therefore, it can be assumed that the conditions of use were similar (ca. several dozen km per day).

However, before the examination of the compatibility of distributions is carried out, the distribution function or the reliability function of the empirical distribution is determined using the Kaplan-Meier method and then the parameters of the hypothetical distributions are estimated using the maximum likelihood estimation (MLE) method [2,23,24,25]. After estimating the hypothetical distributions, the statistics of compliance of the fit of individual hypothetical distributors to the empirical distribution are determined. Based on this the distributions are ranked. If only the assumptions are met, then the rankings of the consistency of distributions are made independently according to three criteria, using the modified Kolmogorov-Smirnov statistics, mean absolute deviation and the value of the logarithmic likelihood function [11,26,27]. The proposed methodology for testing the compliance of distributions is described in detail in [28].

The rest of the paper is organized as follows: in Section 2, the construction, application, causes of damage of the boot lid contactor, as well as the characteristics of the research group are presented. In Section 3, probability distribution of fitting of failure data was determined. Section 4 shows the main results contains data interpretation of boot lid contactor, optimal maintenance strategy, as well as own solution to the problem, which may be used in the future by the vehicle manufacturer. Finally, Section 5 concludes the paper and presents our final remarks.

## 2. Subject of Research

The damage analysis was carried out on a boot lid contactor located in the trunk lid of a medium-class vehicle, marked symbolically D. Its task is to send an electric impulse by pressing it, on the basis of which the boot lock is unlocked and the boot can be opened. The research was carried out on a fleet of 61 segment D passenger cars, produced by one of the leading passenger car manufacturers and used by one of the operators. The tested vehicles were produced in 2009–2015, while all the damages occurred between 2016 and 2018. The tested contactors were mounted in two different types of body configuration of the same vehicle model, i.e., hatchback and estate car.

**Figure 1:**
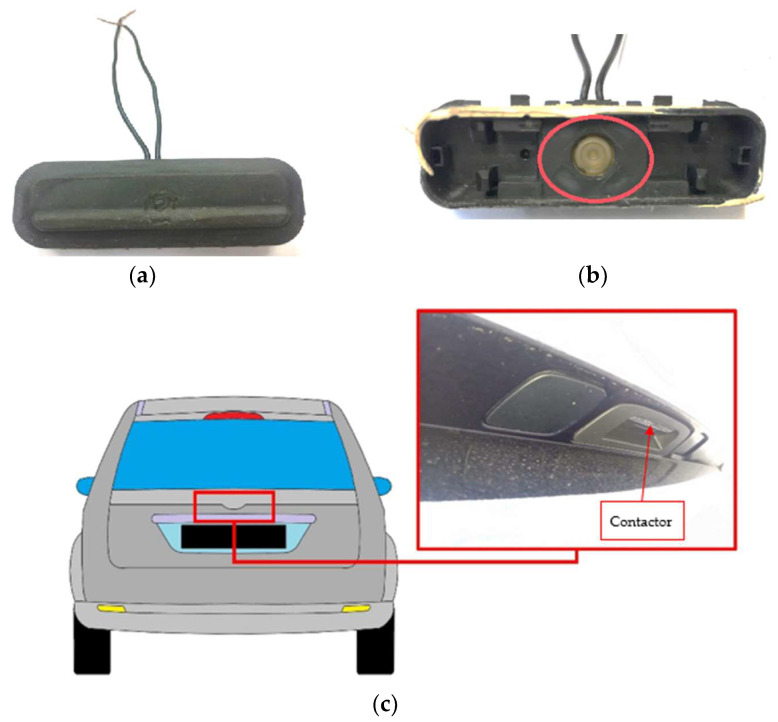
Boot lid contactor; (**a**) front site view, (**b**) front site with an open cover-electronic switch system-button, (**c**) the place where the contactor is located.

The data used for the analysis was expressed as mileage in kilometers of vehicles. All vehicles were used in similar conditions, i.e., the same road infrastructure, similar weather conditions and the same schedule and scope of servicing (scheduled maintenance), in accordance with the vehicle manual. Figure 1 shows a view of the tested element, and the contactor system is marked with a red line. In the studied two-year period of vehicle operation, 41 contactor failures were recorded in the group of 61 and one of all used vehicles.

**Figure 2:**
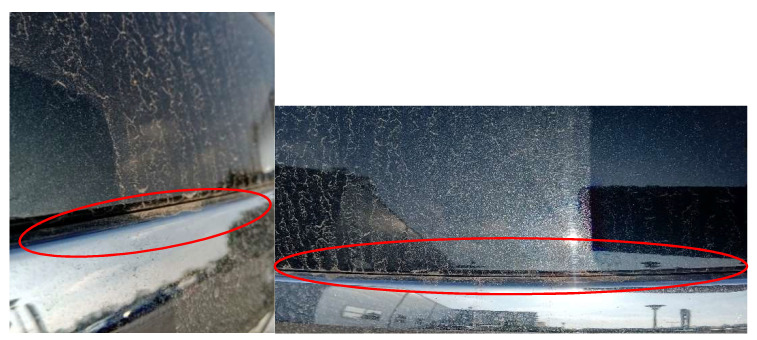
View of the gap between the chrome strip and the vehicle body, through the water entered, causing damage to the boot lid contactor.

The vehicle manufacturer installed a chrome strip in the boot lid (Figure 2), which was to serve as a decorative element and to prevent scratches formed on the paintwork during vehicle operation.

During the use of vehicles, a problem with opening the boot lid was found, regardless of the mileage. The boot lid did not open when the button was pressed to actuate the contactor. At the preliminary stage of the study, the analysis of the causes of damage to the boot lid contactor led to the conclusion that the main factor causing the damage is water entering the contactor. During rain or snowfall, the water penetrated under the decorative strip on the boot lid, under which the contactor was directly located. This was due to a design defect. The manufacturer, at the stage of designing and manufacturing the vehicle, did not provide an adequate seal both between the chrome strip and the vehicle body, and between the strip and the contactor responsible for opening the boot lid. The described damage was included as a design error, because at the design stage, the contactor was not adequately protected against moisture penetrating from the outside. During the warranty period of the vehicle, the costs of corrective maintenance were incurred by the supplier of the vehicle fleet and the repair was performed at an authorized service center. However, after the warranty period, because the described element is not responsible for active or passive safety in the vehicle, the repair was carried out at the cost of the vehicle owner and amounted to 53.73 EUR, which included replacement of the contactor with a new one and the cost of replacement. When the contactor was not damaged, additional security of the contactor was carried out by applying an appropriate sealing in the form of a tape, which wraps the wires entering the contactor. This resulted in effective sealing of the connection and no possibility of water getting onto the contactor control board. The cost of preventive maintenance was 22.02 EUR.

## 3. Failure Data and Probability Distribution Fitting

As example of life data for 2 years of operation for contactor is shown in the Table 1, includes exact failure time (in kilometers) and suspension time (in kilometers). Suspension time is right censored data that did not fail by the end of the test and in all the studied cases, the suspected failure mechanism is design error. Data are divided into two type of body car H–hatchback and E–estate.

The Reliasoft software (HBM Prenscia, London, UK) used in calculation can provide guidance in selecting a distribution based on statistical tests which was calculated for every body type car (hatchback and estate) independently [29,30]. It uses three factors in order to rank distributions: the Kolmogorov-Smirnov (K-S) test, a normalized correlation coefficient (rho) and the likelihood value (LKV).The first factor is a modified Kolmogorov-Smirnov (K-S) test, which is only used to determine the fit of a continuous distribution with known parameters [28,31]. It measures statistical difference between the expected and obtained results and can be performed such that the null and alternative hypotheses are:

- H_0_: the distribution represents the data,
- H_1_: the distribution does not represent the data.

The K-S test statistic ($D_{max}$) is the maximum difference between the observed and predicted probability:

(1) $$D_{max}=\underset{1\leq i\leq n}{max}\left|S_{i}-Q_{i}\right|$$

where $D_{max}$—value of the statistic, n—number of observations, $Q_{i}$—observed probability and $S_{i}$—predicted probability based on the distribution

It should be pointed that observed probability is calculated using median ranks and the difference between those two values is calculated and the largest absolute difference is $D_{max}$.

The modified K-S test determines the probability that the limit value of the $D_{CRIT}$—taken from the tables is smaller than the maximum $D_{max}$ obtained from the calculations:

(2) $$P\left(D_{CRIT}<D_{max}\right)$$

A large $D_{max}$ statistic indicates that there is a significant difference between the theoretical and empirical distributions. The null hypothesis is rejected when the calculated $D_{max}$ value is greater than or equal to critical value $D_{crit}$ at the previously selected significance level. Critical values $D_{crit}$ for the K-S test for different distributions is tabulated in statistical textbooks. The greater the value of the $D_{max}$, the more important is the difference between the hypothetical distribution expressed by the distributor $S_{i}$ and the empirical distribution expressed by the distributor $Q_{i}$. For the final estimate of the critical value of $D_{crit}$, the arithmetic mean denoted as $\hat{D_{CRIT}}$ is taken. Finally, the value of the K-S criterion for assessing the compliance of distributions takes the form:

(3) $$K\text{-}S=100\cdot P\left(\hat{D_{CRIT}}<D_{max}\right)\;$$

Large values of K-S test close to 1, indicate that there is a significant difference between the theoretical distribution and the data set. Hence, the hypothetical distribution is the better the smaller the value of the K-S test.

Second factor is the correlation coefficient test denote rho measures how well the plotted points fit a straight line [31,32]. In this statistic test, the mean absolute deviation of the hypothetical from the empirical distribution is tested, and the statistics used to conformity assessment are determined according to the following formula:

(4) $$rho=100\frac{1}{n}\overset{n}{\underset{i=1}{\sum }}\left|S_{i}-Q_{i}\right|$$

where n—number of observations, $Q_{i}$—observed probability and $S_{i}$—predicted probability based on the distribution.

Third factor called Likelihood value test (LKV) computes the value of the log-likelihood function, given the parameters of the distribution [28,31,32]. The basic idea of this method is to obtain the most likely values of the parameters, for a given distribution, that will best describe the data. The likelihood function *L* depends on continuous random variables $T_{1},T_{2},\ldots ,T_{n},\;S_{1},S_{2}\ldots S_{m}$ which represents the data (observed failures and suspension respectively) and unknown parameters which need to be estimated $\theta_{1},\theta_{2},\ldots ,\theta_{k}$. Because in this case, the likelihood function needs to be expanded to take into account the suspended contactors, the likelihood function is given by:

(5) $$L\left(\theta_{1},\theta_{2},\ldots ,\theta_{k}|T_{1},T_{2},\ldots ,T_{n},\;S_{1},S_{2}\ldots S_{m}\right)=\overset{n}{\underset{i=1}{\prod }}f\left(T_{i};\theta_{1},\theta_{2},\ldots ,\theta_{k}\right)\cdot \overset{m}{\underset{j=1}{\prod }}\left[1-F\left(S_{j};\theta_{1},\theta_{2},\ldots ,\theta_{k}\right)\right]\;$$

where L—the likelihood function, n—observed failures at *T_1_,T_2_,...,T_n_*, *m*—number of suspended data points at *S_1_,S_2_,...,S_m_*, k—the number of estimated parameters, $T_{i}$—failure time of the *i*-th component, *S_j_*—suspension of the *j*-th component, $\theta_{1},\theta_{2},\ldots ,\theta_{k}$—*k* unknown parameters which need to be estimated, $f\left(T_{i};\theta_{1},\theta_{2},\ldots ,\theta_{k}\right)$—probability density function pdf and $F\left(S_{j};\theta_{1},\theta_{2},\ldots ,\theta_{k}\right)$—cumulative density function cdf.

It is often mathematically easier to manipulate this function by first taking the logarithm of it. This log-likelihood function Λ then has the form for right censored data:

(6) $$L\Lambda =\ln L=\overset{n}{\underset{i=1}{\sum }}\ln f\left(T_{i};\theta_{1},\theta_{2},\ldots ,\theta_{k}\right)+\overset{m}{\underset{j=1}{\sum }}\ln\left[1-F\left(S_{j};\theta_{1},\theta_{2},\ldots ,\theta_{k}\right)\right]$$

In the equation above, the first summation is for complete data, the second summation is for right censored data. The maximum likelihood estimators (or parameter values) of $\theta_{1},\theta_{2},\ldots ,\theta_{k}$ are obtained by maximizing *L* or Λ. To define the estimators of the unknown parameters, partial derivatives of the function Λ are determined with respect to the parameters $\theta_{e}$, e=1, 2,…,k. This is done by taking the partial derivative of the log-linear equation for each parameter and setting it equal to zero:

(7) $$\frac{\partial \Lambda }{\partial \theta_{e}}=0,\;e=1,2,\ldots ,\;k\;$$

Next it ranks the selected distributions in terms of the fit to the data entered. In order to determine the ranking, the three tests are used in conjunction with weights assigned to each test. In the Table 2 and Table 3, the second column, contains values obtained using the Kolmogorov-Smirnov test (K-S). The third column, provides the results of the second test, which is a normalized correlation coefficient (rho). The fourth column contains the likelihood values (LKV).

Next received factors obtained from the test (Table 2 and Table 3) are weighted and then summed into one overall WDV (weighted decision variable) value which is given by [15]:

WDV = (K-S Rank × K-S Weight) + (rho Rank × rho Weight) + (LKV Rank × LKV Weight)(8)

In Table 4 and Table 5 the fifth column contains WDV values and the sixth column contains the ranks of the distribution.

The distribution with the lowest WDV value is considered to be the best fit for the data. Software allows user-specified different weights depending on whether the parameter estimation method is rank regression or MLE [31]. In these studies the MLE method was used and the weights assigned to each test were K-S–40%, rho–10% and LKV–50%. The assigned weights for each test are based on engineering practice resulting from the advantages and disadvantages of each of them. The first factor Kolmogorov-Smirnov test has the advantages that the distribution of statistic does not depend on cumulative distribution function being tested and the test is exact. It has the disadvantage that it is more sensitive to deviations near the centre of the distribution than at the tails. The second factor rho test is not sensitive to local deviations but takes into account the global diversity of distributions and is a good supplement to the K-S test. The likelihood function for the suspended data helps demonstrate some of the advantages that MLE analysis has over other parameter estimation techniques. First of all, it takes into account the values of the suspension times, as was showed in the Equation (5). Other tests only take into account the relative location of the suspensions, not the actual time-to-suspension values. This makes MLE a much more powerful tool when dealing with data sets that contain a relatively large number of suspensions.

Details of the weight calculation and choice have been taken from [32] in the reference list. The sum of the three weights for each parameter estimation method must equal 100%. More detailed information about the procedure algorithm and the calculation methods used can be found in the paper [28]. The sixth column in Table 4 and Table 5 contains the model distribution rankings which are ranked according to how well they fit the data, with rank 1 being the best fit; in some cases more than one choice is proposed (Table 4 and Table 5).

For the data contained in Table 1 regarding two types of body (estate and hatchback) during nine years of operation of the vehicles fleet, the 3-P Weibull distribution was identified as the best-fitting for both of them. This is reflected in the last column of Table 4 for hatchback and 5 for estate body type.

The 3-parameter Weibull pdf is given by [3]:

(9) $$f\left(t;\beta ,\eta ,\gamma \right)=\frac{\beta }{\eta }{\left(\frac{t-\gamma }{\eta }\right)}^{\beta -1}e^{-{\left(\frac{t-\gamma }{\eta }\right)}^{\beta }},\;t\geq \gamma ,\;\beta >0,\;\eta >0,\;\gamma \in \mathbb{R}$$

where *f(t)*—density function, *η*—scale parameter, *β*—shape parameter and *γ*—location parameter.

For both types body cars 3-Weibull goodness of fit distribution test *p* value is greater than significance level α = 0.05 and is equal 0.9999 for hatchback and 0.9708 for estate and it can also be noticed that K-S = 1 *p* value.

## 4. Results Analysis

The analysis method uses the maximum likelihood estimation (MLE) for estimating the parameters of the chosen distribution. Rank method uses the median ranks (MED), Confidence bounds method uses the Fisher matrix (FM) [32]. In this case for analyzed contactor, the 3P-Weibull distribution is the suggested model because is highest ranked both for estate and hatchback type car. The following figures compare the fit of the distributions to the probability grids, the curves of the probability distribution function and the probability distribution density function for the tested contactor, divided into two types of car body (estate and hatchback). The calculated parameters for the selected distributions are also given.

### 4.1. Data Interpretation

**Figure 3:**
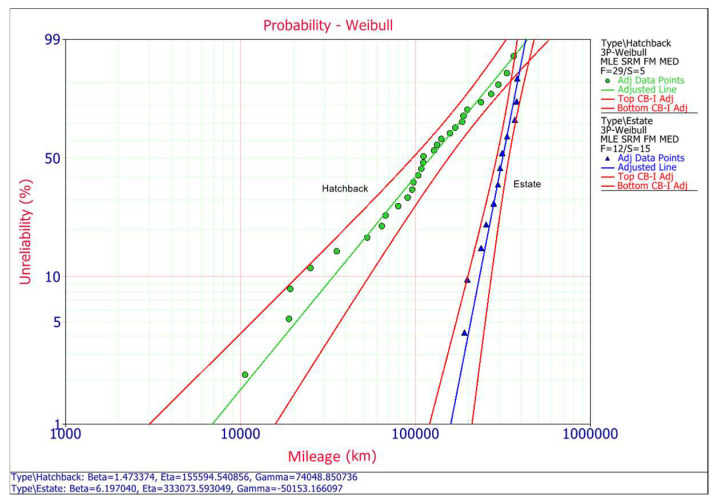
Comparison of 3P-Weibull probability.

The following graphs show blue colour for estate car type and green colour data for the hatchback. The probability plot (Figure 3) shows the trend in the probability of failure over time (expressed in kilometers) and it determines the statistical difference between two populations (estate and hatchback). This allows to compare life distributions from two alternate back body designs (estate and hatchback), in order determine whether the contactor in the first group (estate) will outlast the units in the second (hatchback). Based on this analysis, it was also determine that they have different MDBF parameter (mean distance between failures). For these analysed contactors, most of the failures in the hatchback body type occurred between 85,000 and 272,000 km. In the case of the estate body type the failure was recorded between 140,000 km and 330,000 km.

**Figure 4:**
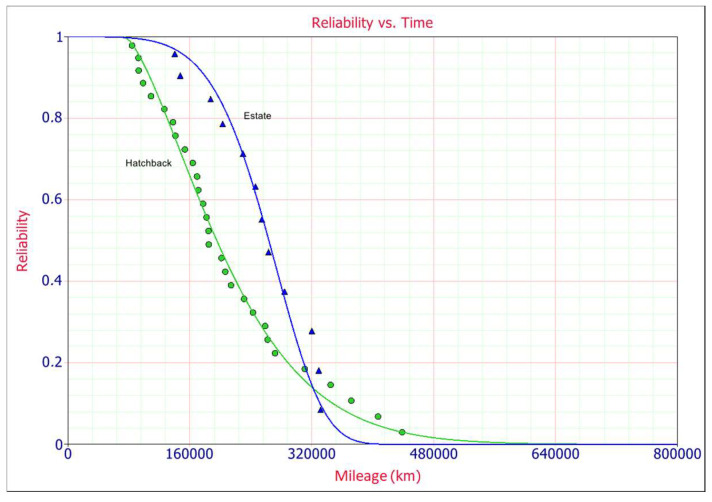
Comparison of 3P-Weibull reliability.

The mean time to failure for hatchbacks is MDBF = 133,487 km and for the estate case it is MDBF = 161,181 km. It also can be noticed that both the beta and eta parameters are different for both car types. For estate body cars the slope is 6197, which is relatively high and for hatchbacks it is 1473, and the eta parameters are *η* = 155,594 km and *η =* 333,074 km, respectively. Failure rate for estate body cars is *h* = 0.0000165/km and for hatchbacks *h* = 0.0000198/km. The reliability values are quite different at different times (Figure 4), at the MDBF of 200,000 km for the hatchback type body over 77% of the contactors are expected to fail, while for estate type body only 4% of the contactors are expected to fail.

**Figure 5:**
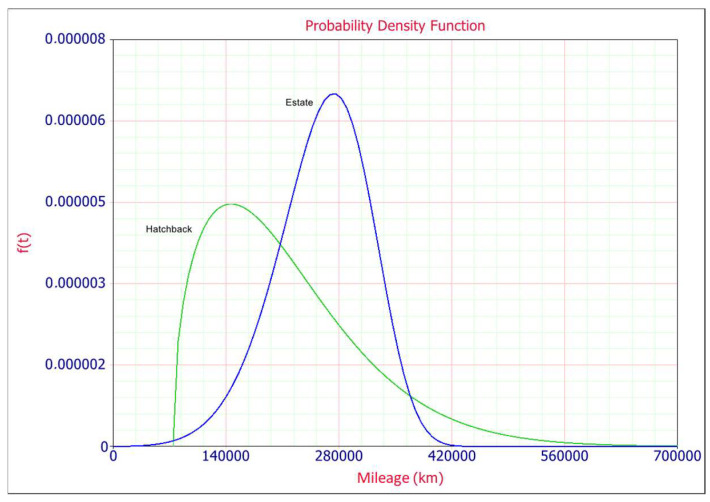
Probability density plot.

The probability density function plot (PDF) is shown in Figure 5 and it is observed that the probability of a contactor failure for estate cars is greater than in the case of hatchback type. In both cases the shape parameter *β* is greater than one and the positively skewed Weibull PDF for hatchback explains that the probability of failure rate increases with time. Location parameter *γ* provides an estimate of the earliest time-to-failure of the units under test and it equals 74.050 km for hatchback and −50.153 km for estate car type. This parameter represents a period without failures, it represents a period of time for which the reliability is 100%. Negative scale parameter in case of estate car type means that the distribution starts at the location *γ* to the left of the origin, it means that the unadjusted line for gamma is concave up.

**Figure 6:**
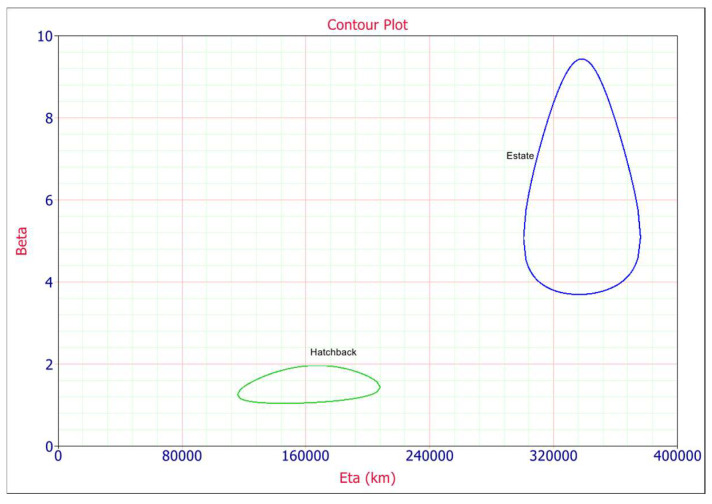
Contour plot at the 90% confidence level.

Contour plot (Figure 6) is used to investigate the probability of occurrence of one failure mode with respect to another failure mode at different percentage of confidence level. Contour plot can be best used to study the inter-dependency of the two types of failures [33]. It is observed that the estate and hatchback type body car failure data do not overlap at 90% confidence level. Hatchbacks (green color plot) show drastically different Weibull characteristics then estate type cars, indicative of an entirely different failure mode. This confirms that the failures of the two components are not dependent at these confidence levels. Separation between contours signifies the populations are statistically different at the specified level of confidence.

### 4.2. Optimal Maintenance Strategy

Trying to determine preventive replacement time maintenance, it must be taken into account that replacing a component before it fails (preventive action) may, under certain circumstances, make better economic sense than replacing the component after it fails (corrective action). Two requirements must be met in order for the preventive replacement of a contactor to be appropriate. First, the total cost of the replacement must be greater after failure than before and second, the failure rate of the contactors must be increasing [1]. Both of them are met, as the cost of planned maintenance is 22.02 EUR and for corrective maintenance it is 57.73 EUR. The price of the corrective maintenance of the boot lid contactor was estimated on the basis of data from several authorized car repair workshops. The amount of 57.73 EUR consists of the price of the purchased contactor 22.02 EUR and the remaining cost of the replacement. In second case, the shape parameter *β* is greater than one for both type of body car and for estate *β* = 6197 and for hatchback *β* = 0.473, thus it means that the failure rate increases with time [34]. To determine the optimum time for such a preventive maintenance action (replacement), we need to mathematically formulate a model that describes the associated costs and risks. In developing the model, it is assumed that if the unit fails before time t, a corrective action will occur and if it does not fail by time t, a preventive action will occur. In other words, the unit is replaced upon failure or after a time of operation, t, whichever occurs first. Thus, the optimum replacement time can be found by minimizing the cost per unit time, *C(t)* is given by [35,36]:

(10) $$C\left(t_{p}\right)=\frac{Total\;Expected\;Replacement\;Cost\;per\;Cycle}{Expected\;Cycle\;Lenght}=\frac{C_{p}\cdot R\left(t_{p}\right)+C_{f}\cdot \left[1-R\left(t_{p}\right)\right]}{\int_{0}^{t_{p}}R\left(s\right)ds}\;$$

where: $C\left(t_{p}\right)$—is the total cost per unit time, $C_{p}$—is the cost of a planned (preventive) replacement, $C_{f}$—is the cost of an unplanned (corrective) replacement, $R\left(t_{p}\right)$—is the reliability of the component at time $t_{p}$ and $t_{p}$—optimal time interval for preventive replacement once the item has reached a specific age.

The optimum replacement time interval, $t_{p}$, is the time that minimizes $C\left(t_{p}\right)$. Hence, the optimum replacement time can be obtained by solving for $t_{p}$:

(11) $$\frac{\partial \left[C\left(t_{p}\right)\right]}{\partial_{t_{p}}}=0$$

**Figure 7:**
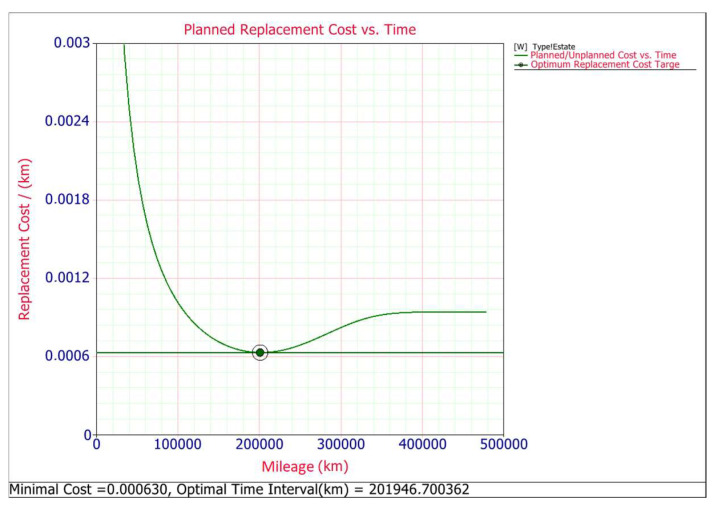
Planned replacement cost for estate.

**Figure 8:**
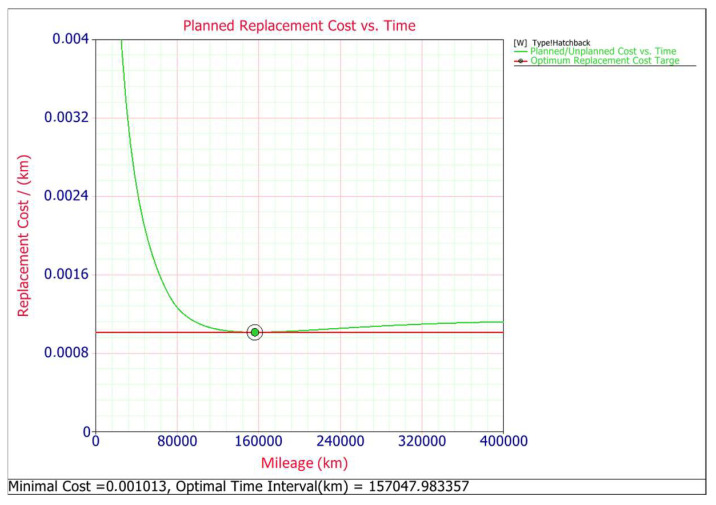
Planned replacement cost for hatchback.

The optimum preventive maintenance time and corresponding cost per unit time are shown graphically for both car body types in Figure 7 and Figure 8. These plots show the optimal replacement interval schedule for minimizing the cost in the long term. The results show that the optimal replacement interval is about 202,000 km for the estate car type and the minimal cost is about 0.000139 EUR per kilometer, while for the hatchback body type the optimal replacement interval 157,000 km, and minimal cost is about 0.000223 EUR per kilometer.

In both plots (Figure 7 and Figure 8) it can be seen that the corrective replacement costs will increase as time increases. The preventive replacement costs will decrease as the time interval increases because as more time passes, fewer preventive replacement actions will need to be performed. The total cost is the sum of these two costs. At one point (time t), a minimum cost point exists that determines the optimum preventive replacement time for the component.

In the analyzed period, i.e., from 2009–2015, 14,971 hatchbacks and 8336 station wagons were registered in Poland, which gives a total of 23,307 cars (Table 6) of all registered cars in this period.

Assuming, in a very simplified manner, that the tested sample (approximately 66.7% of damaged vehicles) reflects the amount of damage in the entire vehicle population, it can be assumed that the cost of unplanned maintenance, in line with the optimal time interval of hatchback cars, would be 349,540 EUR, while for the estate version it is 155,998 EUR. The total cost of unscheduled service of all tested cars registered in the years 2009–2015 is 505,538 EUR (Table 7).

In the absence of any action and failure to comply with optimal cost replacement, assuming that the corrective maintenance takes place after the contactor breaks down and amounts to 5373 EUR the cost of repairing the hatchbacks would be 536,249 EUR and for the estate cars 298,589 EUR??? it would be 834,839 EUR. Thus, the total difference (savings) between the sum of repair costs calculated in accordance with the minimum cost maintenance and without it, amounts to 329,510 EUR.

### 4.3. The Method of Solving the Problem

**Figure 9:**
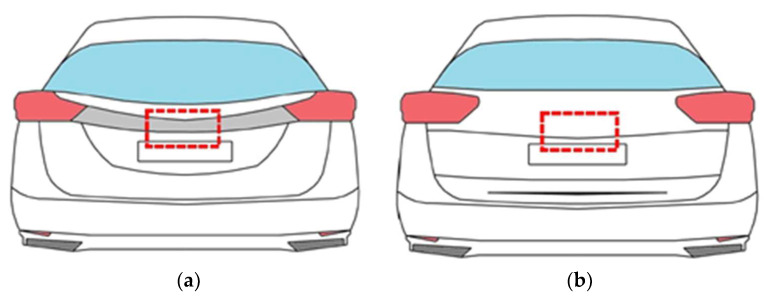
Diagram showing the place of installation of the boot lid contactor (marked with a red line); (**a**) vehicle before modification (has a chrome strip), (**b**) vehicle after modification (without the strip).

Because the main cause of damage to the boot lid contactors was water penetrating under the decorative strip, the design of the trunk lid had to be modified in relation to the original version. The chrome strip was removed from the boot lid. This modification in order to eliminate the cause of the damage is schematically shown in Figure 9. This solution was also used by the manufacturer. As a result, it was impossible for water to enter the contactor, i.e., the main factor causing the damage to this element was eliminated.

## 5. Conclusions

The analysis of the selection of the distribution for the boot lid contactor presented in the paper, carried out with the use of the likelihood value (LKV) test to determine the rank of distributions, is an important argument for the possibility of using the proposed method of forecasting vehicle damage times in the automotive industry. The performed forecasts confirmed that there are methods and technical possibilities helping to accurately forecast the reliability of components of complex technical objects, i.e., motor vehicles. Correct determination of the times to failure may reduce the costs associated with the operation of the vehicle fleet, because by predicting the moment of failure, appropriate preventive measures can be taken. On the other hand, the identification of the causes of the damage may lead to the introduction of appropriate design changes in order to eliminate the damage in the future. It is also possible to give appropriate countermeasures to prevent damage, i.e., to apply a better seal between the top cover of the contactor and the body element to which it is attached.

As the data on which the forecast is built come directly from the actual operating system, the obtained forecasting results take into account the conditions and methods of use prevailing in a specific system of vehicle operation. The benefits resulting from the analysis may be significant both for the fleet operator and the vehicle manufacturer, due to the possibility of determining the costs generated by not using the real durability of the tested component (taking preventive actions too early) as well as losses due to damage and downtime resulting from the repair (no preventive measures are taken).

The obtained results of the selection of the distribution were intended to indicate possible procedures useful for forecasting reliability in the automotive industry. They can be used to assess the accuracy of forecasting and the applicability of the tested procedures in real vehicle operation systems. Forecasting the reliability processes during operation allows for a better understanding of the causes of deterioration of the reliability condition of vehicles, and at a further stage of the research enables to estimate the costs associated with corrective service.

## Figures and Tables

**Table 1:** Failure/suspension date of contactor (F/S–failure, suspension, car body type: H–hatchback, E–Estate).

F/S	Mileage [km]	Car Body Type	F/S	Mileage [km]	Car Body Type	F/S	Mileage [km]	Car Body Type
F	52,621	H	F	126,639	E	F	272,945	H
F	57,801	H	F	128,676	H	S	21,147	E
F	58,001	H	F	133,390	H	S	21,169	E
F	61,628	H	F	143,159	E	S	21,772	E
F	68,100	H	F	144,041	H	S	23,071	E
F	79,051	H	F	151,230	H	S	27,165	H
F	85,974	H	F	153,345	E	S	36,019	E
F	87,451	E	F	158,420	E	S	40,208	E
F	88,050	H	F	161,200	H	S	44,374	H
F	92,003	E	F	163,141	H	S	45,895	E
F	95,636	H	F	163,952	E	S	49,990	E
F	102,178	H	F	169,230	H	S	63,422	E
F	105,600	H	F	176,965	E	S	63,519	H
F	106,639	H	F	193,500	H	S	86,527	H
F	110,558	H	F	199,263	E	S	99,784	E
F	113,359	H	F	204,898	E	S	120,111	E
F	115,000	H	F	206,460	E	S	128,523	E
F	115,210	H	F	214,521	H	S	142,302	E
F	116,762	E	F	231,403	H	S	151,209	E
F	125,489	H	F	253,241	H	S	164,287	E
						S	171,356	H

**Table 2:** Values of factors for hatchback body type.

Distribution	(K-S)	(rho)	LKV
1P-Exponential	98.6344	13.157	−373.271
2P-Exponential	46.7258	5.121	−357.329
Normal	36.3504	5.114	−360.985
Lognormal	0.00136	2.168	−357.914
2P-Weibull	13.3673	3.803	−359.422
3P-Weibull	0.0004	2.316	−356.841
Gamma	1.6526	2.682	−358.212
G-Gamma	0.0018	2.170	−357.914
Logistic	10.3364	3.544	−361.249
Loglogistic	0.00697	2.172	−358.661
Gumbel	58.8010	7.855	−366.116

**Table 3:** Values of factors for estate body type.

Distribution	(K-S)	(rho)	LKV
1P-Exponential	91.569	17.371	−160.982
2P-Exponential	95.994	20.586	−151.111
Normal	0.594	4.003	−146.373
Lognormal	0.020	4.846	−147.220
2P-Weibull	2.379	3.807	−146.021
3P-Weibull	2.918	3.758	−146.001
Gamma	0.016	4.626	−146.840
G-Gamma	9.291	6.338	−143.857
Logistic	0.328	3.580	−146.749
Loglogistic	0.002	3.919	−147.255
Gumbel	5.040	4.615	−146.121

**Table 4:** Ranks of distributions for hatchback body type.

Distribution	K-S	rho	LKV	WDV	Ranking
3P-Weibull	1	4	1	130	1
Lognormal	2	1	4	290	2
G-Gamma	3	2	3	290	2
Loglogistic	4	3	6	490	3
Gamma	5	5	5	500	4
2P-Exponential	9	9	2	550	5
2P-Weibull	7	7	7	700	6
Logistic	6	6	9	750	7
Normal	8	8	8	800	8
Gumbel	10	10	10	1000	9
1P-Exponential	11	11	11	1100	10

**Table 5:** Ranks of distributions for estate body type.

Distribution	K-S	rho	LKV	WDV	Ranking
3P-Weibull	7	2	2	400	1
2P-Weibull	6	3	3	420	2
Logistic	4	1	6	470	3
Normal	5	5	5	500	4
Gamma	2	7	7	500	4
G-Gamma	9	9	1	500	4
Loglogistic	1	4	9	530	5
Gumbel	8	6	4	580	6
Lognormal	3	8	8	600	7
1P-Exponential	10	10	11	1050	8
2P-Exponential	11	11	10	1050	8

**Table 6:** Number of registered cars in 2009–2015 in Poland.

Body Type	Year of Registration							Sum
	2009	2010	2011	2012	2013	2014	2015	
Hatchback	2402	3876	2418	1705	1207	1766	1597	14,971
Estate	538	1379	1338	1131	1084	1817	1049	8336

**Table 7:** Minimal cost replacement.

Type of Body Car	Minimal Replacement Cost [EUR]	Optimal Time Interval [km]	Cost/Per Car [EUR]	Cost for all Cars (2009–2015) [EUR]
Hatchback	0.000223	157,047.98	35.02	349,540
Estate	0.000139	201,946.70	28.07	155,998
			Total cost	505,538

## Data Availability

The data presented in this study are available on request from the corresponding author.
